# The persistence of anti-Spike antibodies following two SARS-CoV-2 vaccine doses in patients on immunosuppressive therapy compared to healthy controls—a prospective cohort study

**DOI:** 10.1186/s12916-022-02587-8

**Published:** 2022-10-05

**Authors:** Ingrid Egeland Christensen, Ingrid Jyssum, Anne Therese Tveter, Joseph Sexton, Trung T. Tran, Siri Mjaaland, Grete Birkeland Kro, Tore K. Kvien, David John Warren, Jørgen Jahnsen, Ludvig A. Munthe, Espen A. Haavardsholm, John Torgils Vaage, Gunnveig Grødeland, Fridtjof Lund-Johansen, Kristin Kaasen Jørgensen, Silje Watterdal Syversen, Guro Løvik Goll, Sella Aarrestad Provan

**Affiliations:** 1 Center for treatment of Rheumatic and Musculoskeletal Diseases (REMEDY), Diakonhjemmet Hospital, P.O Box 23, Vinderen, N-0319 Oslo, Norway; 2 Institute of Clinical Medicine, Faculty of Medicine, University of Oslo, Oslo, Norway; 3 Department of Immunology, Oslo University Hospital, Oslo, Norway; 4 Norwegian Institute of Public Health, Oslo, Norway; 5 Department of Microbiology, Oslo University Hospital, Oslo, Norway; 6 Department of Medical Biochemistry, Oslo University Hospital, Oslo, Norway; 7 Department of Gastroenterology, Akershus University Hospital, Lørenskog, Norway; 8 KG Jebsen Centre for B cell Malignancies, Institute of Clinical Medicine, University of Oslo, Oslo, Norway; 9 ImmunoLingo Convergence Center, University of Oslo, Oslo, Norway; 10 Department of Public Health and Sport Sciences, Inland Norway University of Applied Sciences, Elverum, Norway

**Keywords:** SARS-CoV-2 vaccine, COVID-19, Serologic response, Rheumatic diseases, Inflammatory bowel disease

## Abstract

**Background:**

The durability of vaccine-induced humoral immunity against SARS-CoV-2 in patients with immune-mediated inflammatory diseases (IMIDs) on immunosuppressive therapy is not known. The aim of this study was to compare the persistence of anti-Spike antibodies following two-dose SARS-CoV-2 vaccination between IMID patients and healthy controls and to identify factors associated with antibody decline.

**Methods:**

IMID patients on immunosuppressive medication enrolled in the prospective observational Nor-vaC study were included. Participants received two-dose SARS-CoV-2 vaccination. Serum collected at two time points following vaccination (first assessment within 6–48 days, second within 49–123 days) were analyzed for antibodies binding the receptor-binding domain (RBD) of the SARS-CoV-2 Spike protein. Multivariable regression models estimated percent reduction in anti-RBD over 30 days and factors associated with reduction.

**Results:**

A total of 1108 patients (403 rheumatoid arthritis, 195 psoriatic arthritis, 195 spondyloarthritis, 124 ulcerative colitis, 191 Crohn’s disease) and 134 controls provided blood samples within the defined intervals (median 19 days [IQR 15–24] and 97 days [87–105] after second vaccine dose). Antibody levels were lower in patients compared to controls at both time points, with median anti-RBD 2806 BAU/ml [IQR 1018–6068] in patients and 6187 BAU/ml [4105–7496] in controls (*p*<0.001) at first assessment, and 608 BAU/ml [IQR 58–1053] in patients and 1520 BAU/ml [979–3766] in controls (*p*<0.001) at second assessment. At second assessment, low anti-RBD antibody levels (defined as <200 BAU/ml) were found in 449 (41%) patients, and 6 (5%) controls (*p*<0.001). The change was − 83% in patients and − 66% in controls (*p*<0.001). Patients had a greater estimated 30 days percent reduction in anti-RBD levels compared to controls − 4.9 (95% CI − 7.4 to − 2.4), (*p*<0.05). Among therapies, mono- or combination treatment with tumor necrosis factor inhibitors was associated with the greatest decline.

**Conclusions:**

Within 4 months after vaccination, antibody levels declined considerably in both IMID patients and controls. Patients had lower initial antibody levels and a more pronounced decline compared to healthy controls and were therefore more likely to decline to low antibody levels. These results support that IMID patients need additional vaccine doses at an earlier stage than healthy individuals.

**Supplementary Information:**

The online version contains supplementary material available at 10.1186/s12916-022-02587-8.

## Key messages

1. Anti-Spike antibody decline 4 months post-vaccination was significantly larger in patients (− 83%) compared to controls (− 66%).
2. Four months post-vaccination, antibody levels decreased to a low level (<200 BAU/ml) in 41% of IMID patients and 5% of controls.
3. Among therapies, the use of tumor necrosis factor inhibitors in mono- or combination therapy was associated with the most pronounced decline in anti-Spike antibodies.

## Background

We rely on efficient vaccines for long-lasting protection against COVID-19 disease in order to counter the SARS-CoV-2 pandemic. SARS-CoV-2 vaccines have a proven efficacy in the general population, and antibody levels correlate to the degree of clinical protection against COVID-19 disease [1–3]. However, recent data suggest that patients with immune-mediated inflammatory disease (IMID) treated with certain immunosuppressants have an impaired serological response following vaccination, with lower anti-Spike antibody levels than the general population [4–15]. The impairment of the immune response varies between the different immunosuppressive therapies, where some seemingly do not affect the humoral response, while other drugs, such as rituximab and abatacept, have been found to profoundly reduce vaccine response [12, 15–17].

IMIDs encompass a number of prevalent chronic diseases, including inflammatory joint diseases such as rheumatoid arthritis (RA), psoriatic arthritis (PsA), spondyloarthritis (SpA), and inflammatory bowel disease (IBD); ulcerative colitis (UC); and Crohn’s disease (CD). Immunosuppressive medication, including tumor necrosis factor inhibitors (TNFi), non-TNFi biologic agents, metabolite inhibitors, and targeted small molecule drugs, are pivotal in the treatment of IMIDs [18–21]. Use of immunosuppressive therapies combined with a dysregulated immune system and an increased frequency of several co-morbidities increases the susceptibility to serious infections and vulnerability to adverse outcomes of infectious diseases in these patients [22–24]. There are, however, disparities between therapies regarding the degree of impact on the immune responses and thus the susceptibility to serious infections [12, 15, 17, 25, 26].

Evidence of a waning immune response over time is emerging in the healthy population, both in terms of antibody levels and protection against symptomatic COVID-19 disease [27–35]. Given the risk of severe COVID-19 disease faced by the IMID population [36–38], a possible weakened long-term immunological protection elicited by SARS-CoV-2 vaccines has been raised as a concern for this patient population. A recent study on patients with rheumatic diseases has reported a three-fold decrease in antibodies 6 months after two SARS-CoV mRNA vaccines [39], but the impact of various medications is unknown. Further, it is not known whether there is a difference in the perseverance of vaccine-induced immunity against SARS-CoV-2 between IMID patients and the general population. Data on the long-term effectiveness of the SARS-CoV-2 vaccines in immunosuppressed patients with IMIDs are needed to assess the protection against severe COVID-19 disease over time in this large at-risk population and to make decisions on the appropriate timing of booster doses.

The main aim of this study was to evaluate the persistence of the serologic response to two-dose SARS-CoV-2 vaccination in IMID patients and healthy controls within 4 months post-vaccination. Secondary aims were to compare the rate of decay of anti-Spike antibodies in patients and controls and to identify predictors of antibody decline across vaccine types and immunosuppressive regimens.

## Methods

### Study design and participants

The present study uses data from the ongoing Nor-vaC study (*Nor*wegian study of *va*ccine response to *C*OVID-19 vaccines in patients using immunosuppressive medication within rheumatology and gastroenterology). Nor-vaC is a prospective, observational study which included patients diagnosed with RA, PsA, SpA, UC, or CD who intended to receive SARS-CoV-2 vaccination. The study is conducted at two Norwegian hospitals; the Division of Rheumatology and Research at Diakonhjemmet Hospital (DH) and the Department of Gastroenterology at Akershus University Hospital (AHUS), both with large specialist clinics. Adult patients (aged ≥18 years) were recruited into Nor-vaC prior to the initiation of the Norwegian national vaccination program in February 2021. Eligible patients treated with immunosuppressive drugs were identified from hospital records at DH and AHUS and invited to participate in the study. Eligibility criteria are described in the Additional file 1: Section 1. Health care workers from DH and AHUS constituted the healthy control group. In the present study, we included patients and healthy controls who provided blood samples at both first (6–48 days) and second assessment (49–123 days) after the second vaccine dose, for details of inclusion, see Additional file 1: Fig. S1. Immunosuppressive medications were arranged into the following categories: Metabolite inhibitors (methotrexate, sulfasalazine, leflunomide, azathioprine and mercaptopurine), interleukin inhibitors (tocilizumab, iksekizumab, ustekinumab, secukinumab, risankizumab), vedolizumab (IBD patients only), abatacept (RA patients only), janus kinase inhibitors (JAKi), tumor necrosis factor inhibitors (TNFi) monotherapy, TNFi combination therapy (combined with a metabolite inhibitor or vedolizumab), and rituximab (RA patients only).

The study was approved by an independent ethics committee (Regional Committees for Medical and Health Research Ethics South East, reference numbers 235424, 135924) and institutional review boards. All patients and healthy controls signed informed consent. The study is registered at clinialtrials.gov (NCT04798625).

### Study procedures

According to the national vaccination program, as instructed by the Norwegian Institute of Public Health (NIPH) and administered by the public health system, all participants were vaccinated with two doses of SARS-CoV-2 vaccine, with the exception of those with prior COVID-19 disease who received one vaccine dose only. Initially, there were three SARS-CoV-2 vaccines available: BNT162b2, mRNA-1273, and ChAdOx1, until ChAdOx1 was withdrawn from the Norwegian vaccination program in March 2021 due to reports of serious side effects [40]. The interval between two doses of the mRNA vaccines was 3–6 weeks. All participants who had received one dose of ChAdOx1 vaccine were given one of the mRNA vaccines as the second dose after an interval of 9–12 weeks.

### Data collection

Data was collected using electronic questionnaires handled by Services for Sensitive Data (TSD) at DH and Viedoc version 4 at AHUS. For patients; demographic data, medication use, disease activity, and information regarding previous COVID-19 were collected before vaccination. Disease activity was assessed by the Harvey-Bradshaw index and the Mayo Score in CD and UC patients, respectively and by patient-reported global assessment of disease activity for patients with RA, PsA and SpA. Age, gender, vaccine type, and COVID-19-related information were collected for healthy controls. Participants were asked to self-report COVID-19 disease at follow-up questionnaires. COVID-19 disease was defined by positive PCR and/or rapid antigen test. In addition, participants were linked by a unique personal identification number to the Norwegian Immunisation Registry (SYSVAK) and Norwegian Surveillance System for Communicable Diseases (MSIS) providing information on the date and type of vaccination received and the date of COVID-19 disease when applicable [41, 42].

### Serological analyses

Participants were requested to donate serum samples 2–4 weeks and 3 months after the second vaccine dose. The Department of Immunology at OUH performed the antibody assessments. An in-house bead-based method was used to measure antibodies to the receptor-binding domain (RBD) at the full-length SARS-CoV-2 Spike protein and binding of the Spike-protein to the ACE-2 receptor. This method was validated against a micro-neutralization assay [43]. Results were given in Binding Antibody Units (BAU) per ml.

Antibody levels were categorized according to the official specifications at Oslo University Hospital as follows: less than 5 BAU/ml was defined as negative, 5–19 BAU/ml very weak positive, 20–199 BAU/ml weak positive, 200–1999 BAU/ml positive, 2000–8999 BAU/ml strong positive and 9000 BAU/ml or more very strong positive. 200 BAU/ml was defined as the threshold value for a positive result as 200 BAU/ml was determined to be the lower threshold for detection of neutralizing antibodies by the usage of a micro-neutralization assay [43].

### Outcomes

The main outcome of this study was the persistence of antibodies after two-dose SARS-CoV-2 vaccination in IMID patients and controls, measured as the magnitude of the immunoglobulin G antibody levels to the receptor binding domain on the SARS-CoV-2 spike protein (anti-RBD) at 4 months. Additional outcomes were the percentage reduction in antibodies from first to second assessment in patients compared to controls.

### Statistical analyses

Anti-RBD levels, estimated percentage change in anti-RBD levels, and time interval between first and second assessment of antibody levels were compared between groups using Student’s *t*-test and Mann-Whitney *U* test as appropriate. Participants with increasing anti-RBD levels between first and second serologic assessment post-vaccination were excluded from further analyses (Additional file 1: Fig. S1).

#### Multivariable linear regression

Factors associated with the 30 days estimated percent reduction in anti-RBD levels were identified by entering the following variables in three separate multivariable regression models including all participants; patients vs. controls, types of vaccination, and type of immunosuppressive medication. Diagnoses were entered into a fourth model that only included patients with a type of immunosuppressive medication entered as a possible confounder. Groups of immunosuppressive medications with less than 30 patients were excluded from the regression analyses. All models were corrected for age, gender, anti-RBD at first assessment, and time between blood samples. For details, see Additional file 1: Fig. S2 and Table S1.

The relationship between body mass index (BMI) and measures of disease activity and percent change in anti-RBD was explored in disease-specific models. To explore the impact of age on antibody decline, an age-stratified multivariable regression was performed.

## Results

### Population characteristics

Between 6th of April 2021, and 9th of November 2021, a total of 1108 patients (median age 54 years [IQR 43–64]; 617 women [56%]) and 134 controls (median age 46 years [IQR 35–56]; 111 women [83%]) provided two blood samples within the defined intervals for serologic assessment (median 19 days [IQR 15–24] and 97 days [87–105] after second vaccine dose), and were included in the present study (Additional file 1: Fig. S3). Characteristics of the participants are presented in Table 1.

**Table 1:** Characteristics of the participants

	Controls (***n***=134)	Patients (***n***=1108)	Rheumatoid arthritis (***n***=403)	Psoriatic arthritis (***n***=195)	Spondyloarthritis (***n***=195)	Ulcerative colitis (***n***=124)	Crohn’s disease (***n***=191)
**Demographics**							
Age (years), median (IQR)	45.6 (34.9–56.1)	54.3 (43.2–64.1)	61.2 (51.3–69.1)	57.9 (48.3–64.7)	50.5 (42.0–59.2)	45.7 (33.4–54.6)	43.0 (29.6–53.9)
Female, *n* (%)	111 (83)	617 (56)	308 (76)	104 (53)	75 (38)	53 (43)	77 (40)
Male, *n* (%)	23 (17)	491 (44)	95 (24)	91 (47)	120 (62)	71 (57)	114 (60)
Patient reported disease activity, median (IQR)^ab^		20 (10–40)	20 (10–41)	25 (11–40)	21 (10–40)	15 (5–35)	20 (10–30)
**Vaccines,*n*(%)**							
BNT162b2 × 2	11 (8)	780 (70)	282 (70)	135 (69)	125 (64)	94 (76)	144 (75)
mRNA-1273 × 2	43 (32)	285 (26)	108 (26.8)	52 (27)	60 (31)	25 (20)	40 (21)
Mixed^c^	80 (60)	37 (3)	12 (3)	7 (3.5)	10 (5)	3 (2.4)	5 (3)
COVID-19 infection and one vaccine dose	0	6 (1)	1 (0.2)	1 (0.5)	0	2 (1.6)	2 (1)
**Medication,*n*(% of the total number of patients)**							
Rituximab		31 (3)	31 (8)				
Tumor necrosis factor inhibitors							
- Monotherapy		464 (42)	51 (12.5)	60 (31)	163 (83.5)	67 (54)	123 (64)
- Combination therapy^d^		261 (23.5)	106 (26)	64 (32)	25 (13)	24 (19)	43 (23)
Interleukin inhibitors							
- Tocilizumab		19 (2)	17 (4)	2 (1)			
- Other interleukin inhibitors^e^		25 (2)		6 (3)	2 (1)	3 (2)	14 (7)
Janus kinase inhibitors		22 (2)	13 (3)	2 (1)		6 (5)	1 (1)
Abatacept		6 (0.5)	6 (1.5)				
Vedolizumab		32 (3)				22 (18)	10 (5)
Metabolite inhibitors							
- Methotrexate monotherapy		218 (19.5)	161 (40)	53 (27)	4 (2)		
- Other metabolite inhibitors^f^		17 (1.5)	11 (3)	4 (2)		2 (2)	
Prednisolone monotherapy		13 (1)	7 (2)	2 (3)	1 (0.5)		

Patients using prednisolone in doses <10mg/day in combination with other medication groups are included in all groups

^a^Patient reported disease activity at baseline, indicated on a visual analog scale 0–100

^b^Missing information in 23 patients

^c^ChAdOx1 + BNT162b2/mRNA-1273 or BNT162b2 + mRNA-1273

^d^In combination with metabolite inhibitors or vedolizumab

^e^Ustekinumab, risankizumab, secukinumab, iksekizumab

^f^Azathioprine, merkaptopurin, sulfasalazine, leflunomide in monotherapy or in combination with each other or in combination with methotrexate

### Persistence of serological response

**Fig. 1:**
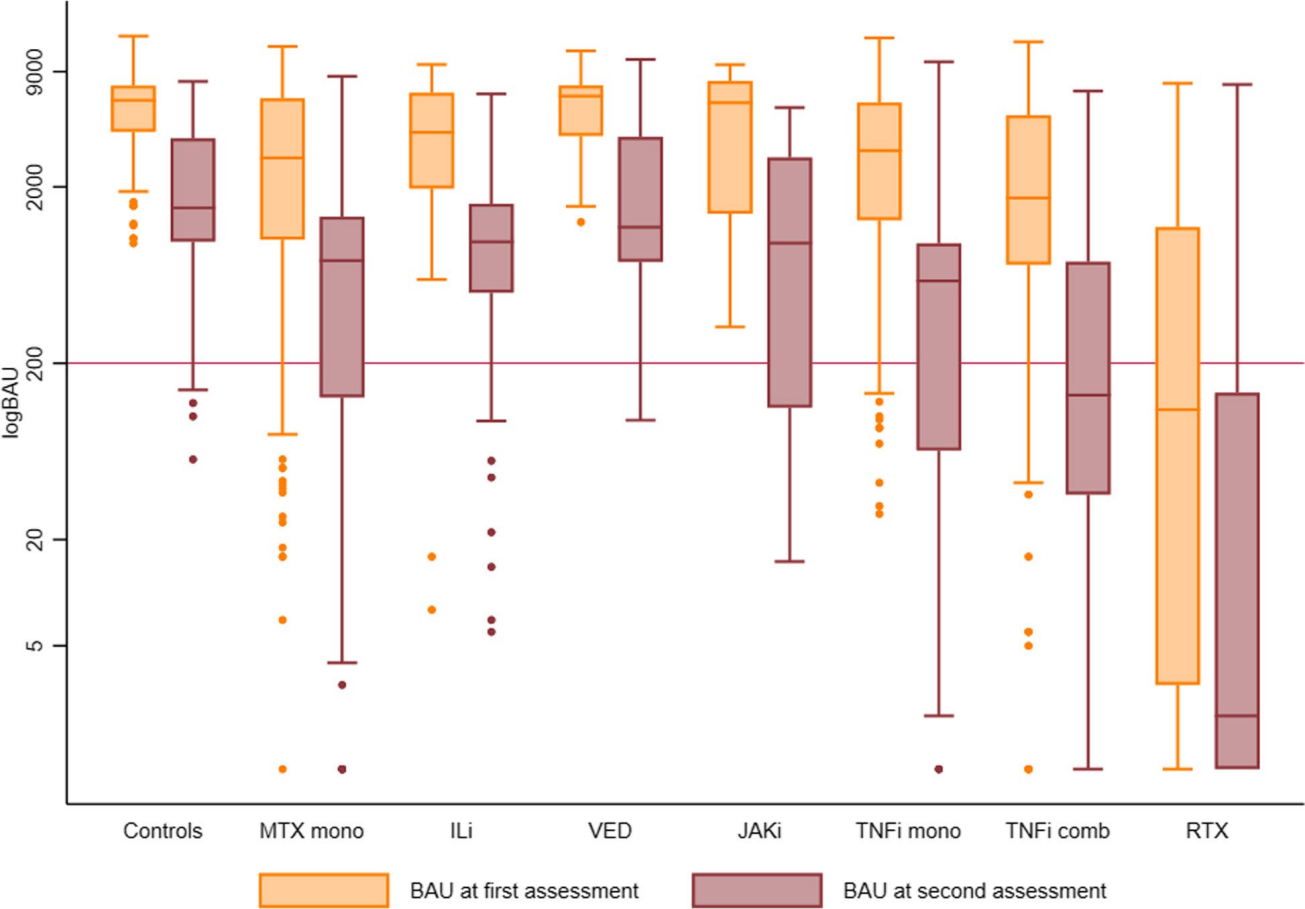
Levels of anti-RBD antibodies at the first and second assessment according to medication group. The orange bars show anti-RBD levels at the first assessment and the purple bars show anti-RBD at the second assessment, 6–48 and 49–123 days after the second vaccine dose, respectively. Bars indicate the lower and upper quartiles. Horizontal lines inside the bars indicate the median. Vertical lines through the bars show the minimum (Q1−1.5×IQR) and maximum value (Q3+1.5×IQR). Dots indicate outliers. A cut-off at 200 BAU/ml is indicated by a red line. MTX mono, methotrexate monotherapy; ILi, interleukin inhibitors including tocilizumab, ustekinumab, iksekizumab, risankizumab, secukinumab; VED, vedolizumab; JAKi, janus kinase inhibitor; TNFi mono, tumor necrosis factor inhibitor in monotherapy; TNFi comb, tumor necrosis factor inhibitor in combination with metabolite inhibitor(s) or vedolizumab; RTX, rituximab. All groups include patients using prednisolone in doses <10mg/day in combination with other medication

Anti-RBD levels were significantly lower in patients as compared to controls at first (median anti-RBD BAU/ml 2806 [IQR 1018–6068] vs. 6187 [IQR 4105–7496], *p* <0.001), and second assessment post-vaccination (median anti-RBD BAU/ml 608 [IQR 58–1053] vs. 1520 [979–3766], *p* <0.001) (Table 2). Changes in anti-RBD levels between the first and second assessment by medication groups and by diagnoses are presented in Fig. 1 and in Additional file 1: Fig. S4, respectively.

**Table 2:** Serological response at first and second assessment following two-dose vaccination in patients and controls

	Controls		Patients	
	First sera assessment	Second sera assessment	First sera assessment	Second sera assessment
Median anti-RBD level (IQR)	6187 (4105–7496)	1520 (979–3766)	2806 (1018–6068)	608 (58–1053)
Median change in anti-RBD level (IQR)^#^	− 3332 (− 5096 to − 2206)		− 2039 (− 4304 to − 806)	
Median percent change in anti-RBD level (IQR)^*^	− 66 (− 79 to − 49)		− 83 (− 94 to − 66)	
Mean (SD) number of days between first and second assessment^##^	75 (16)		75 (17)	
Anti-RBD <5, *n* (%)	0	0	17 (1.5)	56 (5)
Anti-RBD 5–19, *n* (%)	0	0	13 (1)	74 (6.5)
Anti-RBD 20–199, *n* (%)	0	6 (5)	62 (6)	319 (29)
Anti-RBD 200–1999, *n* (%)	10 (7.5)	70 (52)	366 (33)	511 (46)
Anti-RBD 2000–8999, *n* (%)	107 (80)	58 (43)	599 (54)	145 (13)
Anti-RBD ≥ 9000, *n* (%)	17 (12.5)	0	51 (4.5)	3 (0.5)

First sera assessment 6–48 days after second vaccine dose. Second sera assessment 49–123 days after second vaccine dose. Serological response is anti-SARS-CoV-2 IgG antibodies to the receptor binding domain (RBD) measured as *BAU/ml* binding antibody units/ml, *IQR* inter quartile range

^#^Change in median anti-RBD level compared across groups by Mann-Whitney *U* test: *p* <0.001

^*^Percent change in anti-RBD level compared between groups by Mann-Whitney *U* test: *p* <0.001

^##^Mean number of days between first and second assessment compared between groups by Student’s *t*-test *p*=0.77

**Fig. 2:**
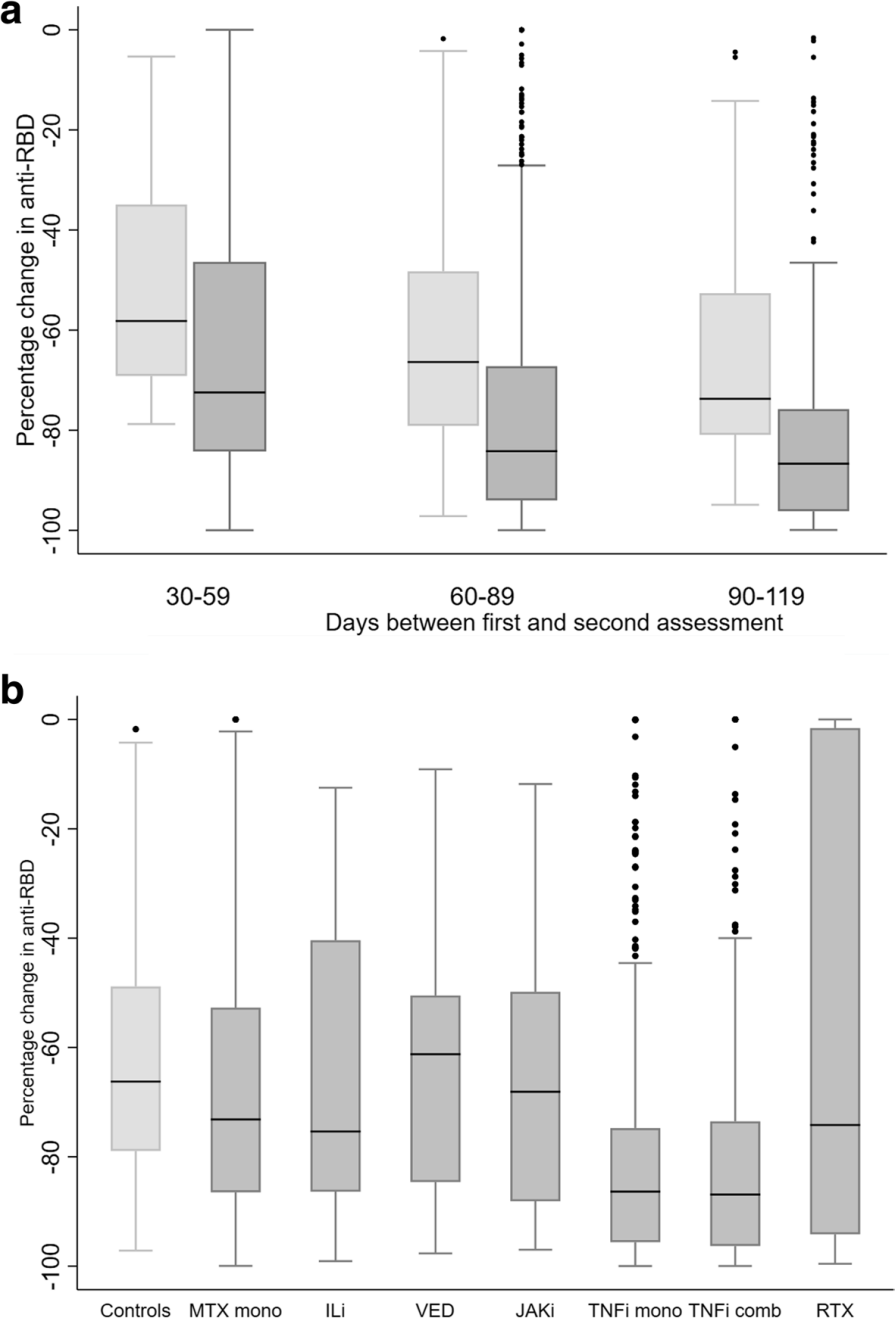
**a** Percentage change in anti-RBD levels between the first and second assessment, stratified by an interval of 30 days. **b** Percentage change in anti-RBD levels between first and second assessment according to medication group. **a** The bars show the percentage change in anti-RBD levels stratified by an interval of 30 days for controls (light gray bars) and patients (dark gray bars). **b** The bars show the percentage change in anti-RBD levels between the first and second assessment in controls (light gray bar) and in patients (dark gray bars) according to medication groups. Bars indicate the lower and upper quartiles. Horizontal lines inside the bars indicate the median. Vertical lines through the bars show the minimum (Q1−1.5×IQR) and maximum value (Q3+1.5×IQR). Dots indicate outliers. MTX mono, methotrexate monotherapy; ILi, Interleukin inhibitors including tocilizumab, ustekinumab, iksekizumab, risankizumab, secukinumab; VED, vedolizumab; JAKi, janus kinase inhibitor; TNFi mono, tumor necrosis factor inhibitor in monotherapy; TNFi comb, tumor necrosis factor inhibitor in combination with metabolite inhibitor(s) or vedolizumab; RTX, rituximab. All groups include patients using prednisolone in doses <10mg/day in combination with other medication

The median reduction of anti-RBD was significantly higher in patients (− 83% [IQR − 94 to − 66]) as compared to controls (− 66% [IQR − 79 to − 49]), *p*<0.0001 (Table 2, Fig. 2a). As presented in Fig. 2b, the median change was greatest in the TNFi mono (− 86% [IQR − 96 to − 75], *p*<0.001) and TNFi combination (− 87% [IQR − 96 to − 74], *p* <0.001) groups compared to controls. The percent reduction in anti-RBD was significantly larger for all diagnoses, compared to controls, Additional file 1: Table S2.

**Fig. 3:**
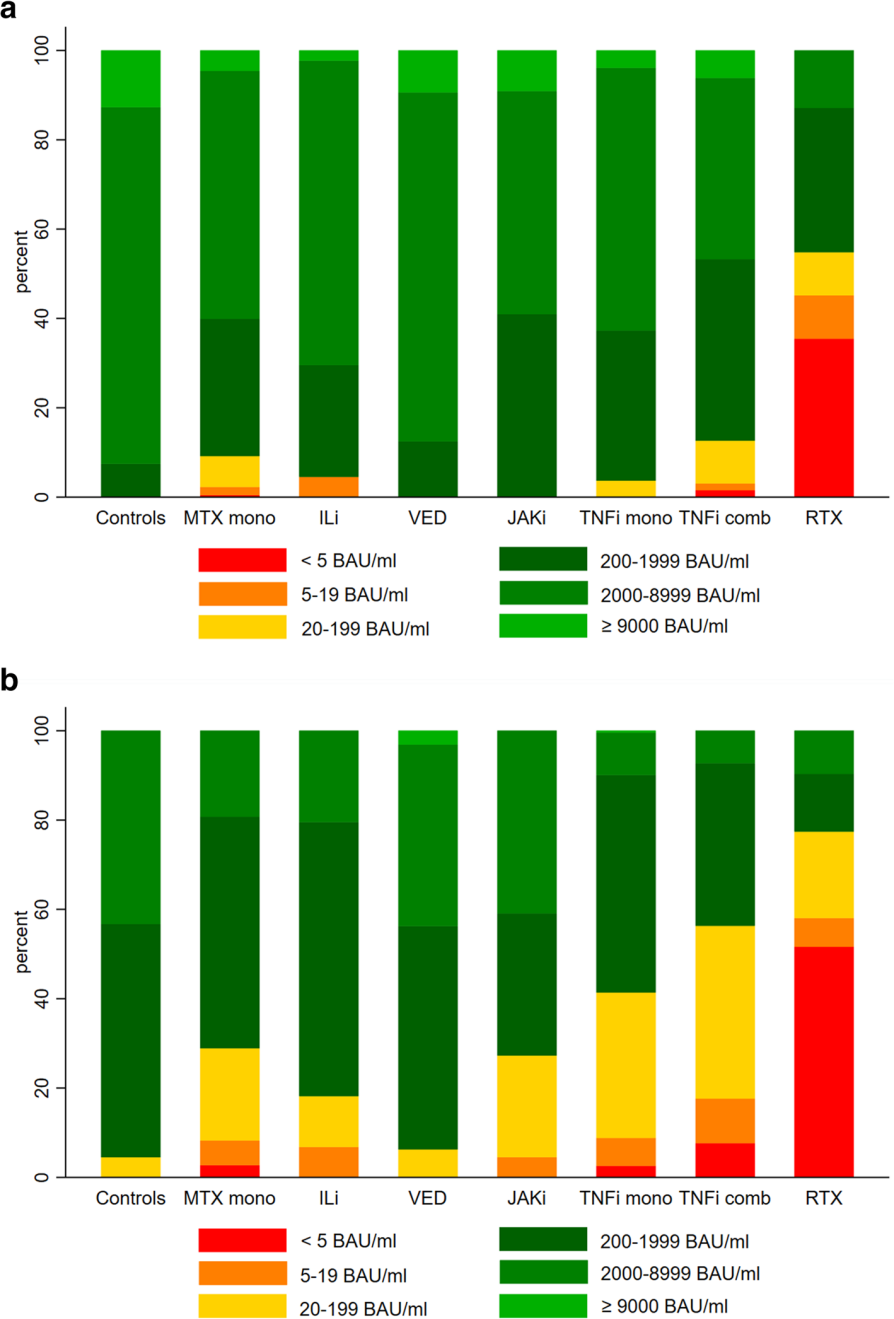
Percent distribution of anti-RBD levels at the first (**a**) and second (**b**) assessment in patients and healthy controls. **a** Percent distribution of anti-RBD levels at the first assessment 6–48 days after the second vaccine dose in controls and in patients according to medication groups. **b** Percent distribution of anti-RBD levels at the second assessment 49–123 days after the second vaccine dose in controls and in patients according to medication group. MTX mono, methotrexate monotherapy; ILi, interleukin inhibitors including tocilizumab, ustekinumab, iksekizumab, risankizumab, secukinumab; VED, vedolizumab; JAKi, janus kinase inhibitor; TNFi mono, tumor necrosis factor inhibitor in monotherapy; TNFi comb, tumor necrosis factor inhibitor in combination with metabolite inhibitor(s) or vedolizumab; RTX, rituximab. All groups include patients using prednisolone in doses <10mg/day in combination with other medication

At second assessment, 449 (41%) patients vs. 6 (5%) controls had low anti-RBD levels (<200 BAU/ml), *p*<0.0001, whereas only 148 (13.5%) patients vs. 58 (43%) controls had anti-RBD levels > 2000 BAU/ml, *p*<0.001 (Table 2). The distribution of anti-RBD levels by medication groups at first and second assessment are shown in Fig. 3a, b and in Additional file 1: Table S3. In patients treated with TNFi in mono- or combination therapy, 192 (41%) and 147 (56%) had anti-RBD < 200 BAU/ml at the second assessment, respectively (Fig. 3a, b, Additional file 1: Table S3). Only 46 (10%) and 19 (7%) of patients using TNFi monotherapy or TNFi combination therapy, respectively, had persisting anti-RBD levels > 2000 BAU/ml at the second assessment. The medication groups with the highest proportion of patients with anti-RBD>2000 BAU/ml at the second assessment were JAKi (9 patients [41%]) and Vedolizumab (14 patients [44%]); however, the number of patients in each of these medication groups were small (*n*=22 and *n*=32) (Fig. 3b). Additional file 1: Fig. S4 shows anti-RBD levels across diagnoses at both assessments. Of all diagnoses, the highest percentage of patients with anti-RBD <200 BAU/ml at second assessment, was found in CD (92 patients [48%]).

### Factors influencing percent reduction in anti-RBD levels

In multivariable regression models (Table 3) a significantly greater estimated 30 days percent reduction in anti-RBD levels was found in patients (β − 6.4 [95% CI − 8.4 to − 4.3]) compared to controls (*p*<0.001). The difference remained significant after adjusting for vaccine type. Age-stratified (< 50 and ≥ 50 years of age) multivariable regression showed similar results with a significantly greater estimated 30 days percent reduction in anti-RBD levels in patients compared to controls for both age categories (data not shown). Use of TNFi in mono- or combination therapy was associated with a larger reduction in anti-RBD levels (*β* − 8.6 [95% CI − 10.7 to − 6.5], *β* − 8.1 [95% CI − 10.4 to − 5.9], *p*<0.001, respectively), compared to controls. The reduction in anti-RBD levels varied significantly across diagnoses. Patients with UC had a more pronounced decline than RA patients (*β* − 2.9 [95% CI − 5.7 to − 0.2], *p*<0.05. When comparing vaccine type using BNT162b2 × 2 as reference, mRNA-1273 × 2 or mixed vaccine type was significantly associated with a lower reduction in antibodies (*β* 4.4 [95% CI 3.0–5.9], *p*<0.001, and *β* 2.8 [95% CI 0.2–5.4], *p*<0.05, respectively).

**Table 3:** Linear regression models of estimated 30 days percent reduction in anti-RBD level

	Model 1 β (95% CI)	Model 2 β (95% CI)	Model 3 β (95% CI)	Model 4 β (95% CI)
**Demographics**				
Age in years	0.0 (-0.0–0.1)	0.1 (-0.0–0.1)	-0.0 (-0.1–0.0)	-0.0 (-0.1–0.0)
Female gender	1.2 (-0.1–2.4)	1.2 (-0.1–2.4)	0.7 (-0.6–1.9)	0.7 (-0.6–2.1)
Patients vs controls	-6.4 (-8.4–-4.3) ^**^	-4.9 (-7.4–-2.4)^*^	-	
**Vaccine type**				
BNT162b2 x 2		-		
COVID-19 infection and vaccine		8.3 (-0.2–16.8)		
Mixed^a^		2.8 (0.2–5.4)^*^		
mRNA-1273 x 2		4.4 (3.0–5.9) ^**^		
**Medication**				
Methotrexate monotherapy			-1.9 (-4.3– 0.5)	-
Interleukin inhibitors			-0.9 (-4.5–2.7)	1.8 (-1.7–5.3)
Vedolizumab			0.7 (-3.3–4.7)	5.1 (0.7–9.5)^*^
TNF mono			-8.6 (-10.7–-6.5) ^**^	-6.2 (-8.4–-4.1)^**^
TNF comb			-8.1 (-10.4–-5.9)^**^	-6.0 (-8.0–-4.0)^**^
Rituximab			-0.3 (-4.8–4.2)	1.0 (-3.3–5.3)
**Diagnosis**				
Rheumatoid arthritis				-
Psoriatic arthritis				0.5 (-1.4–2.5)
Spondyloarthritis				0.8 (-1.4–3.1)
Ulcerative colitis				-2.9 (-5.7–-0.2)^*^
Crohn’s disease				-2.3 (-4.7–0.1)
***R***^**2**^**adjusted**	0.21	0.23	0.26	0.26

Linear regression models. Dependent variable of each model is estimated reduction in anti-RBD level in 30 days

All models are adjusted for anti-RBD levels at first assessment and time between first and second sera assessment. Please see Table S1 for details

Variables selected by forwards stepwise selection

Model 1 includes age, sex and patients vs. controls

Model 2 includes age, sex, patients vs. controls and type of vaccine

Model 3 includes age, sex, type immunosuppressive medication vs controls

Model 4 includes age, sex, type immunosuppressive medication and diagnosis. Controls were excluded from this model due to collinearity. (Methotrexate monotherapy and rheumatoid arthritis are comparators)

^*^p < 0.05, ^**^p < 0.001

^a^ChAdOx1 + BNT162b2/mRNA-1273 or BNT162b2 + mRNA-1273

Age and gender were not associated with the percentage change in anti-RBD levels.

In the disease-specific models (Additional file 1: Tables S4 and S5) markers of disease activity and BMI were not significantly associated with estimated 30 day reduction in anti-RBD levels.

## Discussion

In this large observational study examining the persistence of anti-Spike antibodies after two-dose SARS-CoV-2 vaccination in IMID patients on immunosuppressive therapies, we demonstrated that antibody levels declined considerably in both IMID patients and controls within 4 months after the second vaccine dose. Patients had lower anti-RBD levels at the first assessment, and a more rapid reduction in antibody levels, resulting in a higher proportion of patients with low antibody levels after 4 months compared to controls. The overall reduction of anti-RBD levels within 4 months after the second vaccine dose was significantly higher in patients (− 83%) compared to controls (− 66%). A considerably larger proportion of IMID patients (41%) compared to controls (5%) declined to low (<200 BAU/ml) antibody levels 4 months post vaccination. By medication groups, those treated with rituximab or TNFi mono- or combination therapy were more likely to have low antibody levels at 4 months after the second vaccine dose. Furthermore, a diagnosis of UC was associated with the highest antibody decline over time.

We have previously reported that patients with IMIDs have an attenuated SARS-CoV-2 vaccine response compared to healthy controls at the first assessment after a standard two-dose regimen [12, 15]. We now report continued low antibody levels in this 4-month follow-up study. A threshold antibody level giving actual, clinical protection against breakthrough COVID-19 infection has not yet been established. However, evidence for a correlation between antibody levels and the protective immunity against COVID-19 infection is emerging [3, 44–48]. Gilbert et al. demonstrated a decreasing risk of serious COVID-19 disease with increasing antibody level and that the protection against COVID-19 induced by the mRNA-1273 vaccine was improved with increasing levels of antibodies [3]. Given the considerable decrease in antibody levels demonstrated in this present study and recently reported by others [31, 39], the duration of protection against COVID-19 afforded by the vaccines remains uncertain.

In this current study, we show that anti-RBD levels decrease more rapidly in IMID patients than in healthy controls. Recent studies in the general population have demonstrated waning antibody levels over time and reduced long-term protection against COVID-19 disease induced by SARS-CoV-2 vaccines [27–34]. Antibodies have also been shown to decline following SARS-CoV-2 infections after a peak between 20 and 30 days after onset of symptoms, although most individuals had high levels of IgG up to 94 days after infections [47]. Short-term serological responses have been investigated in patients with autoimmune diseases treated with immunosuppressive medications, demonstrating lower serological response rates and antibody levels after two-dose SARS-CoV-2 vaccination [5, 6, 9, 11, 17, 49]. However, there are limited data available regarding the persistence of humoral immunity in immunosuppressed patients. A recent study by Levin et al. assessing humoral response over 6 months after the second vaccine dose, reported that antibody levels were severely depleted in immunosuppressed patients compared to those without immunosuppressive therapies (only 13 patients were followed until 6 months post-vaccination). In contrast to our study, they found similar rates of antibody reduction between those with and without immunosuppression [31]. The present study demonstrates a greater reduction in antibody levels in patients (83%) than recently found by Frey et al., who reported a 64% reduction in antibodies 6 months after two-dose SARS-CoV-2 vaccination in 326 patients with rheumatic diseases on immunosuppressive therapy [39]; however, no controls were available. A more rapid decline in antibody levels in IMID populations could be due to the continued low level of immunoglobulin production in this population, rather than an increased clearance of antibodies.

Immunity to SARS-CoV-2 also involves cellular immune responses, and evidence of a good T-cell response despite poor humoral response is emerging [12, 50–52]. The association between a persistent humoral immune response and a cellular response has not been fully elucidated. A recent study by Chen et al. followed 27 patients who had recovered from SARS-CoV-2 infection. Although the anti-RBD IgG level had decreased significantly by approximately 7 months post infection, the study reports that the SARS-CoV-2 specific CD4+ T-cell response persisted, with no significant change during the follow-up period [50].

There are some limitations to this present study. Patients were older than controls and the gender distribution for both cohorts was not equal. However, age was not an effect modifier of the association between patients and controls and the rate of antibody decline. The majority of patients received vaccine type BNT162b2, while healthy controls received a combination of one ChAdOx1 and one mRNA vaccine or two mRNA-1273 vaccines. There were differences in the type of immunosuppressive medication prescribed to patient groups. Only RA patients are treated with rituximab, and given the known negative effect of rituximab on the ability to mount a serologic response, the comparison of serologic response between RA and other diagnoses is difficult. We could not fully adjust for channeling bias in the regression models.

The estimation of the percent reduction in anti-RBD levels during 30 days was based on two samples per individual, consequently, the model may not fully capture the changing rate of decay at different time points. However, the time between the first and second assessments was not significantly different between patients and controls. We cannot exclude the possibility of residual confounding due to the study design. Patients who did not provide serum samples at the second assessment were excluded from our analyses. This group may include those who had a high initial anti-RBD response at the first assessment and who therefore had low motivation to provide further serum samples. Participants with increasing anti-RBD antibodies between assessments were excluded from the present study with the assumption that many of these have been exposed to the SARS-CoV-2 virus. However, there is a possibility that some of these participants with increasing antibody levels were late peakers [31]. Additionally, we do not have methods available to identify those previously infected with COVID-19, and we cannot exclude that participants who were subjected to asymptomatic COVID-19 disease before vaccination were wrongly included in the study as COVID-19-naïve. However, the prevalence in Norway was low at that point of time, and the system in place for tracing infection makes this less likely.

To our knowledge, this is the largest study to date assessing the persistence of serologic response after SARS-CoV-2 vaccination in IMID patients treated with immunosuppressive medication compared to healthy controls. The prospective study design and a study population consisting of a large patient cohort and controls are two of the strengths of this study. Sera drawn at two time-points after the second vaccine dose enabled us to study how the vaccine response change over a longer period of time. Further, an important strength is the long time period from the second vaccine dose until the second blood sampling. This distinguishes our study from the majority of previous work on IMID patients that have assessed vaccine response at one time-point closer to the date of the second vaccination. Additionally, the generalizability of our results is increased by the composition of the patient population constituted by several autoimmune diseases from both rheumatology and gastroenterology, allowing assessment of vaccine response across a range of diagnoses and immunosuppressive treatment regimens.

## Conclusions

In conclusion, our results are important when planning vaccine regimens for IMID patients and when prioritizing groups for additional vaccine doses. Our work supports a program of three initial vaccine doses and that further booster doses may be of particular importance in IMID patients who may also need this earlier than the general population.

## Supplementary Information

**Additional file 1: Section 1.** Inclusion and Exclusion Criteria. **Section 2.** Supplementary Figures S1-S4. **Fig. S1.** Flow-chart of analyses population. **Fig. S2.** Margins plot of the estimated 30 days reduction at different intervals between first and second serum assessment. **Fig. S3.** Scatter plot of antibody levels and timing of blood sampling. **Fig. S4.** Levels of anti-RBD antibodies at first and second assessment across diagnoses. **Section 3.** Supplementary Tables S1-S5. **Table S1.** Predictors of anti-RBD level at second assessment. **Table S2.** Median percentage change in anti-RBD level between first and second assessment across diagnoses. **Table S3.** Serological response at first and second assessment by medication group. **Table S4.** Linear regression models of estimated percent reduction in anti-RBD level in 30 days in patients with inflammatory joint diseases. **Table S5.** Linear regression models of estimated percent reduction in anti-RBD level in 30 days in patients with inflammatory bowel diseases.

## Data Availability

Data are available upon reasonable request after we have published all data on our predefined research objectives. The data will only be made available after the submission of a project plan outlining the reason for the request and any proposed analyses and will have to be approved by the Nor-vaC steering group. Project proposals can be submitted to the project leader (gurolovik.goll@diakonsyk.no). Data sharing will have to follow appropriate regulations.

## Abbreviations

AHUS: Akershus University Hospital
BAU: Binding Antibody Unit
CD: Crohn’s disease
DH: Diakonhjemmet Hospital
IBD: Inflammatory bowel disease
IMID: Immune-mediated inflammatory disease
IQR: Inter-quartile range
JAKi: Janus kinase inhibitors
MSIS: Norwegian Surveillance System for Communicable Diseases
NIPH: Norwegian Institute of Public Health
OUH: Oslo University Hospital
PCR: Polymerase chain reaction
PsA: Psoriatic arthritis
RA: Rheumatoid arthritis
RBD: Receptor-binding domain
SD: Standard deviation
SpA: Spondyloarthritis
SYSVAK: Norwegian Immunisation Registry
TNFi: Tumor necrosis factor inhibitors
TSD: Services for sensitive data
UC: Ulcerative colitis

## References

[1] Polack FP, Thomas SJ, Kitchin N, Absalon J, Gurtman A, Lockhart S (2020). Safety and efficacy of the BNT162b2 mRNA COVID-19 vaccine. N Engl J Med.

[2] Baden LR, El Sahly HM, Essink B, Kotloff K, Frey S, Novak R (2021). Efficacy and safety of the mRNA-1273 SARS-CoV-2 vaccine. N Engl J Med.

[3] Gilbert PB, Montefiori DC, McDermott AB, Fong Y, Benkeser D, Deng W (2022). Immune correlates analysis of the mRNA-1273 COVID-19 vaccine efficacy clinical trial. Science (New York, NY).

[4] Haberman RH, Herati R, Simon D, Samanovic M, Blank RB, Tuen M (2021). Methotrexate hampers immunogenicity to BNT162b2 mRNA COVID-19 vaccine in immune-mediated inflammatory disease. Ann Rheum Dis.

[5] Jena A, Mishra S, Deepak P, Kumar-M P, Sharma A, Patel YI (2021). Response to SARS-CoV-2 vaccination in immune mediated inflammatory diseases: systematic review and meta-analysis. Autoimmun Rev.

[6] Geisen UM, Berner DK, Tran F, Sümbül M, Vullriede L, Ciripoi M (2021). Immunogenicity and safety of anti-SARS-CoV-2 mRNA vaccines in patients with chronic inflammatory conditions and immunosuppressive therapy in a monocentric cohort. Ann Rheum Dis.

[7] Furer V, Eviatar T, Zisman D, Peleg H, Paran D, Levartovsky D (2021). Immunogenicity and safety of the BNT162b2 mRNA COVID-19 vaccine in adult patients with autoimmune inflammatory rheumatic diseases and in the general population: a multicentre study. Ann Rheum Dis.

[8] Sakuraba A, Luna A, Micic D (2022). Serologic response to coronavirus disease 2019 (COVID-19) vaccination in patients with immune-mediated inflammatory diseases: a systematic review and meta-analysis. Gastroenterology.

[9] Kennedy NA, Lin S, Goodhand JR, Chanchlani N, Hamilton B, Bewshea C (2021). Infliximab is associated with attenuated immunogenicity to BNT162b2 and ChAdOx1 nCoV-19 SARS-CoV-2 vaccines in patients with IBD. Gut.

[10] Kappelman MD, Weaver KN, Boccieri M, Firestine A, Zhang X, Long MD (2021). Humoral immune response to messenger RNA COVID-19 vaccines among patients with inflammatory bowel disease. Gastroenterology.

[11] Deepak P, Kim W, Paley MA, Yang M, Carvidi AB, Demissie EG (2021). Effect of immunosuppression on the immunogenicity of mRNA vaccines to SARS-CoV-2: a prospective cohort study. Ann Intern Med.

[12] Jyssum I, Kared H, Tran TT, Tveter AT, Provan SA, Sexton J, et al. Humoral and cellular immune responses to two and three doses of SARS-CoV-2 vaccines in rituximab-treated patients with rheumatoid arthritis: a prospective, cohort study. Lancet Rheumatol. 2021. 10.1016/S2665-9913(21)00394-5 PMC8700278 34977602

[13] Braun-Moscovici Y, Kaplan M, Braun M, Markovits D, Giryes S, Toledano K (2021). Disease activity and humoral response in patients with inflammatory rheumatic diseases after two doses of the Pfizer mRNA vaccine against SARS-CoV-2. Ann Rheum Dis.

[14] Boekel L, Steenhuis M, Hooijberg F, Besten YR, van Kempen ZLE, Kummer LY, et al. Antibody development after COVID-19 vaccination in patients with autoimmune diseases in the Netherlands: a substudy of data from two prospective cohort studies. Lancet Rheumatol. 2021;3(11):e778-e88. 10.1016/S2665-9913(21)00222-8 PMC8346242 34396154

[15] Syversen SW, Jyssum I, Tveter AT, Tran TT, Sexton J, Provan SA (2022). Immunogenicity and safety of standard and third-dose SARS-CoV-2 vaccination in patients receiving immunosuppressive therapy. Arthritis Rheumatol (Hoboken, NJ).

[16] Arnold J, Winthrop K, Emery P (2021). COVID-19 vaccination and antirheumatic therapy. Rheumatology (Oxford, England).

[17] Friedman MA, Curtis JR, Winthrop KL (2021). Impact of disease-modifying antirheumatic drugs on vaccine immunogenicity in patients with inflammatory rheumatic and musculoskeletal diseases. Ann Rheum Dis.

[18] Lamb CA, Kennedy NA, Raine T, Hendy PA, Smith PJ, Limdi JK (2019). British Society of Gastroenterology consensus guidelines on the management of inflammatory bowel disease in adults. Gut.

[19] van der Heijde D, Ramiro S, Landewe R, Baraliakos X, Van den Bosch F, Sepriano A (2017). 2016 update of the ASAS-EULAR management recommendations for axial spondyloarthritis. Ann Rheum Dis.

[20] Gossec L, Baraliakos X, Kerschbaumer A, de Wit M, McInnes I, Dougados M (2020). EULAR recommendations for the management of psoriatic arthritis with pharmacological therapies: 2019 update. Ann Rheum Dis.

[21] Smolen JS, Landewé RB, Bijlsma JW, Burmester GR, Dougados M, Kerschbaumer A (2020). EULAR recommendations for the management of rheumatoid arthritis with synthetic and biological disease-modifying antirheumatic drugs: 2019 update.

[22] Listing J, Gerhold K, Zink A (2013). The risk of infections associated with rheumatoid arthritis, with its comorbidity and treatment. Rheumatology (Oxford, England).

[23] Her M, Kavanaugh A (2016). Alterations in immune function with biologic therapies for autoimmune disease. J Allergy Clin Immunol.

[24] Christensen IE, Lillegraven S, Mielnik P, Bakland G, Loli L, Sexton J, et al. Serious infections in patients with rheumatoid arthritis and psoriatic arthritis treated with tumour necrosis factor inhibitors: data from register linkage of the NOR-DMARD study. Ann Rheum Dis. 2022;81(3):398-401. 10.1136/annrheumdis-2021-221007 PMC8862047 34625404

[25] Andersen KM, Bates BA, Rashidi ES, Olex AL, Mannon RB, Patel RC (2022). Long-term use of immunosuppressive medicines and in-hospital COVID-19 outcomes: a retrospective cohort study using data from the National COVID Cohort Collaborative. Lancet Rheumatol.

[26] Boekel L, Stalman EW, Wieske L, Hooijberg F, van Dam KPJ, Besten YR (2022). Breakthrough SARS-CoV-2 infections with the delta (B.1.617.2) variant in vaccinated patients with immune-mediated inflammatory diseases using immunosuppressants: a substudy of two prospective cohort studies. Lancet Rheumatol.

[27] Tartof SY, Slezak JM, Fischer H, Hong V, Ackerson BK, Ranasinghe ON (2021). Effectiveness of mRNA BNT162b2 COVID-19 vaccine up to 6 months in a large integrated health system in the USA: a retrospective cohort study. Lancet (London, England).

[28] Shrotri M, Navaratnam AMD, Nguyen V, Byrne T, Geismar C, Fragaszy E (2021). Spike-antibody waning after second dose of BNT162b2 or ChAdOx1. Lancet (London, England).

[29] Widge AT, Rouphael NG, Jackson LA, Anderson EJ, Roberts PC, Makhene M (2021). Durability of responses after SARS-CoV-2 mRNA-1273 vaccination. N Engl J Med.

[30] Goldberg Y, Mandel M, Bar-On YM, Bodenheimer O, Freedman L, Haas EJ (2021). Waning immunity after the BNT162b2 vaccine in Israel. N Engl J Med.

[31] Levin EG, Lustig Y, Cohen C, Fluss R, Indenbaum V, Amit S (2021). Waning immune humoral response to BNT162b2 Covid-19 vaccine over 6 months. N Engl J Med.

[32] Mizrahi B, Lotan R, Kalkstein N, Peretz A, Perez G, Ben-Tov A (2021). Correlation of SARS-CoV-2-breakthrough infections to time-from-vaccine. Nat Commun.

[33] Doria-Rose N, Suthar MS, Makowski M, O'Connell S, McDermott AB, Flach B (2021). Antibody persistence through 6 months after the second dose of mRNA-1273 vaccine for Covid-19. N Engl J Med.

[34] Khoury J, Najjar-Debbiny R, Hanna A, Jabbour A, Abu Ahmad Y, Saffuri A (2021). COVID-19 vaccine - long term immune decline and breakthrough infections. Vaccine.

[35] Erice A, Varillas-Delgado D, Caballero C (2022). Decline of antibody titres 3 months after two doses of BNT162b2 in non-immunocompromised adults. Clin Microbiol Infect.

[36] Ungaro RC, Brenner EJ, Gearry RB, Kaplan GG, Kissous-Hunt M, Lewis JD (2021). Effect of IBD medications on COVID-19 outcomes: results from an international registry. Gut.

[37] Raiker R, DeYoung C, Pakhchanian H, Ahmed S, Kavadichanda C, Gupta L (2021). Outcomes of COVID-19 in patients with rheumatoid arthritis: a multicenter research network study in the United States. Semin Arthritis Rheum.

[38] Cordtz R, Lindhardsen J, Soussi BG, Vela J, Uhrenholt L, Westermann R (2021). Incidence and severeness of COVID-19 hospitalization in patients with inflammatory rheumatic disease: a nationwide cohort study from Denmark. Rheumatology (Oxford, England).

[39] Frey S, Chiang TP-Y, Connolly CM, Teles M, Alejo JL, Boyarsky BJ, et al. Antibody durability 6 months after two doses of SARS-CoV-2 mRNA vaccines in patients with rheumatic and musculoskeletal disease. Lancet Rheumatol. 2022;4(4):e241-e3. 10.1016/S2665-9913(21)00417-3 PMC8765758 35072108

[40] Pottegård A, Lund LC, Karlstad Ø, Dahl J, Andersen M, Hallas J (2021). Arterial events, venous thromboembolism, thrombocytopenia, and bleeding after vaccination with Oxford-AstraZeneca ChAdOx1-S in Denmark and Norway: population based cohort study. BMJ (Clinical research ed).

[41] Norwegian Institute of Public Health (2021). Norwegian immunisation registry (SYSVAK).

[42] Norwegian Institute of Public Health (2021). Norwegian surveillance system for communicable diseases (MSIS).

[43] Tran TT, Vaage EB, Mehta A, Chopra A, Kolderup A, Anthi A, et al. Titers of antibodies the receptor-binding domain (RBD) of ancestral SARS-CoV-2 are predictive for levels of neutralizing antibodies to multiple variants. BioRxiv 2022.03.26.484261. 10.1101/2022.03.26.484261.

[44] Chia WN, Zhu F, Ong SWX, Young BE, Fong SW, Le Bert N (2021). Dynamics of SARS-CoV-2 neutralising antibody responses and duration of immunity: a longitudinal study. Lancet Microbe.

[45] Khoury DS, Cromer D, Reynaldi A, Schlub TE, Wheatley AK, Juno JA (2021). Neutralizing antibody levels are highly predictive of immune protection from symptomatic SARS-CoV-2 infection. Nat Med.

[46] Cromer D, Steain M, Reynaldi A, Schlub TE, Wheatley AK, Juno JA, et al. Neutralising antibody titres as predictors of protection against SARS-CoV-2 variants and the impact of boosting: a meta-analysis. Lancet Microbe. 2022;3(1):e52-e61. 10.1016/S2666-5247(21)00267-6 PMC8592563 34806056

[47] Seow J, Graham C, Merrick B, Acors S, Pickering S, Steel KJA (2020). Longitudinal observation and decline of neutralizing antibody responses in the three months following SARS-CoV-2 infection in humans. Nat Microbiol.

[48] Bergwerk M, Gonen T, Lustig Y, Amit S, Lipsitch M, Cohen C (2021). Covid-19 breakthrough infections in vaccinated health care workers. N Engl J Med.

[49] Shehab M, Abu-Farha M, Alrashed F, Alfadhli A, Alotaibi K, Alsahli A (2021). Immunogenicity of BNT162b2 vaccine in patients with inflammatory bowel disease on infliximab combination therapy: a multicenter prospective study. J Clin Med.

[50] Chen J, Liu X, Zhang X, Lin Y, Liu D, Xun J (2021). Decline in neutralising antibody responses, but sustained T-cell immunity, in COVID-19 patients at 7 months post-infection. Clin Transl Immunol.

[51] Goel RR, Painter MM, Apostolidis SA, Mathew D, Meng W, Rosenfeld AM (2021). mRNA vaccines induce durable immune memory to SARS-CoV-2 and variants of concern. Science (New York, NY).

[52] Naaber P, Tserel L, Kangro K, Sepp E, Jürjenson V, Adamson A (2021). Declined antibody responses to COVID-19 mRNA vaccine within first three months.

