# Supplementary material for: The persistence of anti-Spike antibodies following two SARS-CoV-2 vaccine doses in patients on immunosuppressive therapy compared to healthy controls—a prospective cohort study
Source: BMC Med. 2022 Oct 5;20:378. doi: 10.1186/s12916-022-02587-8 (PMC9534475; doi:10.1186/s12916-022-02587-8)
Supplement: Supplementary file 1 — Additional file 1: Section 1. Inclusion and Exclusion Criteria. Section 2. Supplementary Figures S1-S4. Fig. S1. Flow-chart of analyses population. Fig. S2. Margins plot of the estimated 30 days reduction at different intervals between first and second serum assessment. Fig. S3. Scatter plot of antibody levels and timing of blood sampling. Fig. S4. Levels of anti-RBD antibodies at first and second assessment across diagnoses. Section 3. Supplementary Tables S1-S5. Table S1. Predictors of anti-RBD level at second assessment. Table S2. Median percentage change in anti-RBD level between first and second assessment across diagnoses. Table S3. Serological response at first and second assessment by medication group. Table S4. Linear regression models of estimated percent reduction in anti-RBD level in 30 days in patients with inflammatory joint diseases. Table S5. Linear regression models of estimated percent reduction in anti-RBD level in 30 days in patients with inflammatory bowel diseases. [file 12916_2022_2587_MOESM1_ESM.docx]

**Additional file 1**

I E Christensen*, I Jyssum*, AT Tveter et al. **The persistence of anti-Spike antibodies following two SARS-CoV-2 vaccine doses in patients on immunosuppressive therapy compared to healthy controls – a prospective cohort study**

Table of Contents

[Section 1. Inclusion and Exclusion Criteria 2](#_Toc115181391)

[Section 2. Supplementary figures 3](#_Toc115181392)

[Section 3. Supplementary tables 7](#_Toc115181393)

# Section 1. Inclusion and Exclusion Criteria

|  | |
| --- | --- |
| **Inclusion Criteria** | - An established clinical diagnosis of one of the following immune-mediated diseases: rheumatoid arthritis (RA), spondyloarthritis (SpA), psoriatic arthritis (PsA), ulcerative colitis (UC), and Crohn’s disease (CD) - On treatment with relevant immunosuppressive and/or immunomodulating medication^*^ - Adult patients (> 18 years) - Patient intends to obtain vaccination against COVID-19 during the next six months |
| **Exclusion Criterion** | - Allergy or intolerance to elements of the COVID-19 vaccines |

*Relevant immunosuppressive medication

| **Medication group** | **Included medications** |
| --- | --- |
| Tumor necrosis factor inhibitor | Infliximab, etanercept, golimumab, adalimumab, certolizumab pegol |
| Janus kinases inhibitor | Tofacitinib, baricitinib, upadacitinib, filgotinib |
| Tumor necrosis factor inhibitor in combination | + methotrexate, azathioprine, sulfasalazine, leflunomide, mercaptopurine or prednisolone |
| Methotrexate |  |
| Azathioprine |  |
| Tocilizumab |  |
| Abatacept |  |
| Sulfasalazine |  |
| Vedolizumab |  |
| Ustekinumab |  |
| Secukinumab |  |
| Iksekizumab |  |
| Leflunomide |  |
| Prednisolone |  |
| Rituximab |  |
| Risankizumab |  |
| 6-mercaptopurine |  |
|  |  |
|  |  |

# Section 2. Supplementary figures

**Figure S1** Flow-chart of analyses population:

**Figure S1 Legend**

*4/62 with increasing anti-RBD between first and second assessment were healthy controls. Median days from second vaccination to first assessment was 18 (IQR 14–26) and 19 (IQR 15–24) for the participants excluded and the participants included, respectively.

**Figure S2** Margins plot of the 30 days estimated percent reduction at different intervals between first and second serum assessment


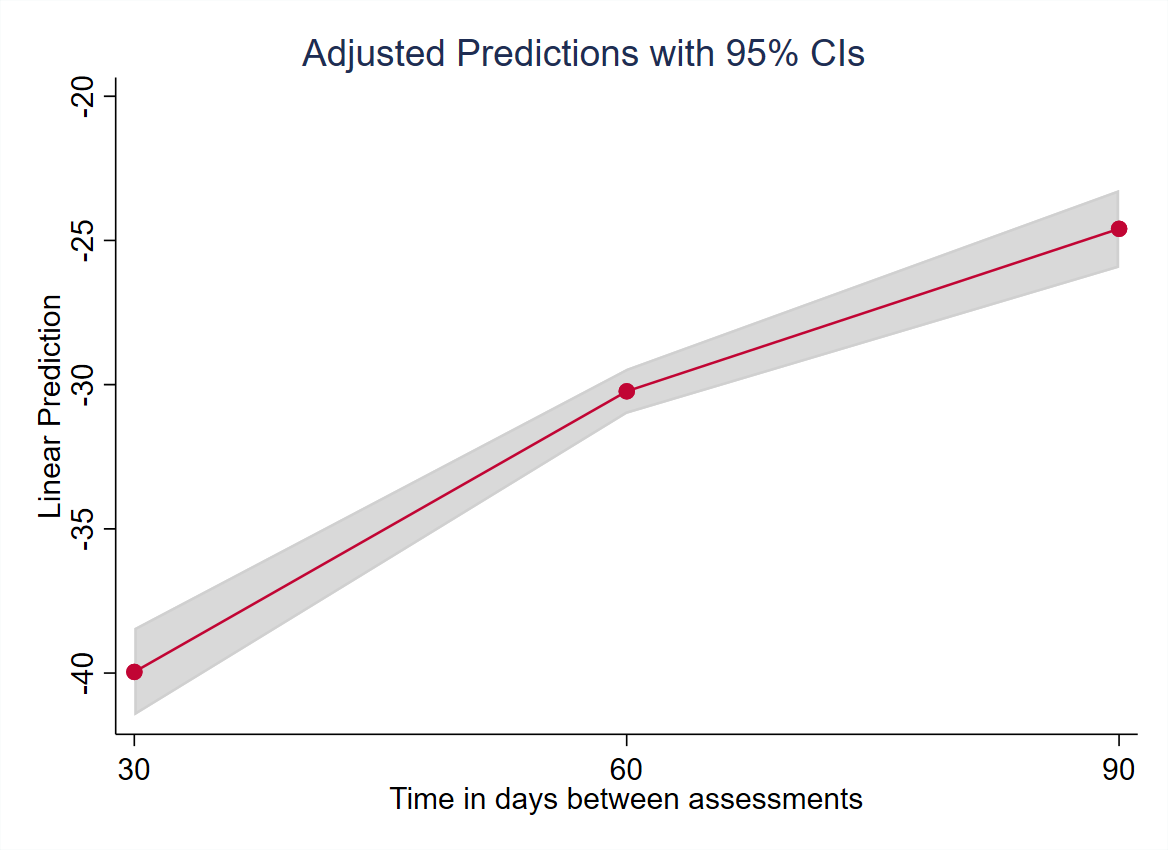


**Figure S2 Legend**

**Construction of the linear regression model for change in anti-RBD over time:**

The estimated percentage reduction in anti-RBD levels standardized to a 30 days interval between first and second assessment was calculated. Figure S2 shows a margins plot of the 30 days estimated percent reduction at different intervals between first and second serum assessment. The linear regression model that best explained the 30 days estimated percent reduction was arrived at by a stepwise forward variable selection comparing different model adjustments for time, including time as continuous and ordinal dummy variables. The models were compared by likelihood ratio test, adjusted R^2^ and Akaike information criteria (Table S1). The model that best captured the rate of decay included tertiles of time between assessments.

**Figure S3** Scatter plots of antibody levels and timing of blood sampling


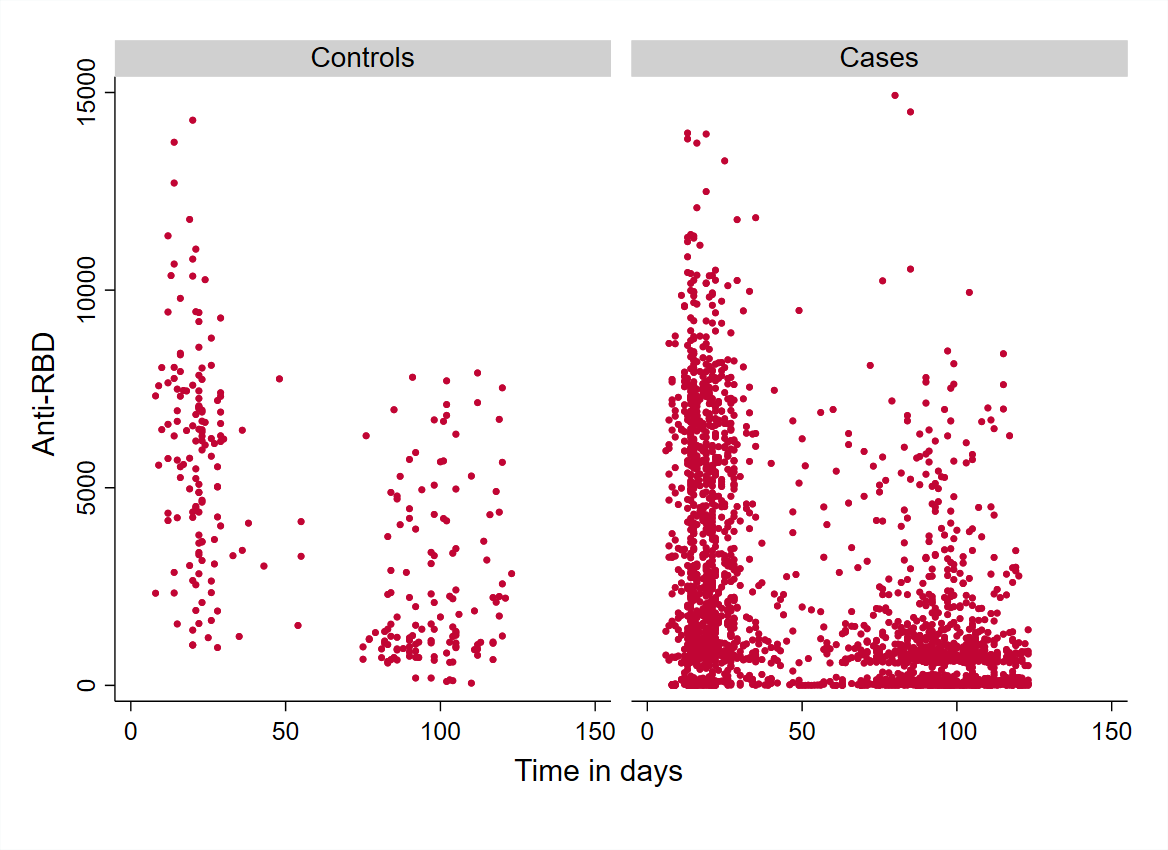


**Figure S3 Legend**

One outlier removed from graph, anti-RBD measured in BAU/ml

**Figure S4** Levels of anti-RBD antibodies at first and second assessment across diagnoses


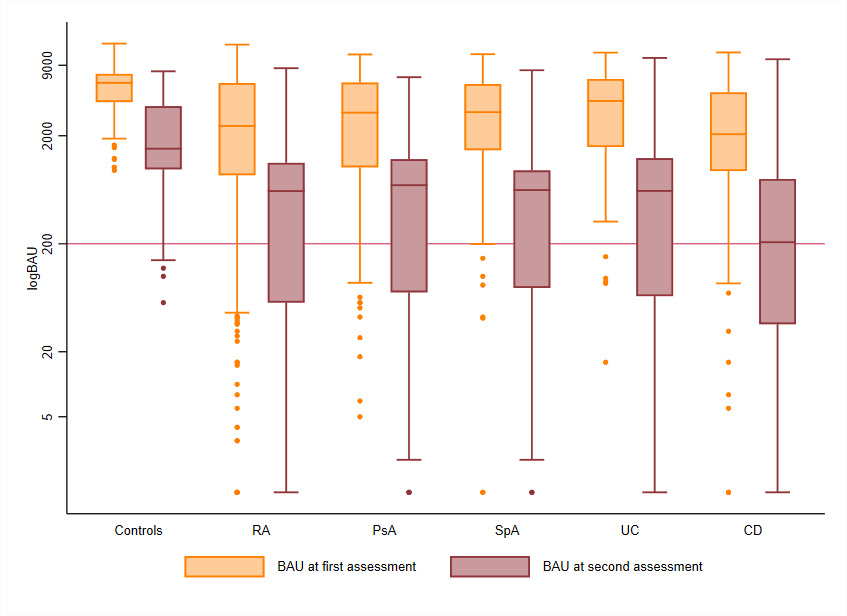


**Figure S4 Legend**

Bars indicate the lower and upper quartiles. Horizontal lines inside the bars indicate the median. Vertical lines through the bars show the minimum (Q1-1.5xIQR) and maximum value (Q3+1.5xIQR). Dots indicate outliers. A cutoff at 200 BAU/ml is indicated by a red line.

MTX mono: methotrexate monotherapy; ILi: Interleukin inhibitors including tocilizumab, ustekinumab, iksekizumab, risankizumab, secukinumab; VED: vedolizumab; JAKi: janus kinase inhibitor; TNFi mono: Tumor necrosis factor inhibitor in monotherapy; TNFi comb: Tumor necrosis factor inhibitor in combination with metabolite inhibitor(s) or vedolizumab; RTX: rituximab. All groups include patients using prednisolone in doses <10mg/day in combination with other medication.

# Section 3. Supplementary tables

**Table S1** Predictors of anti-RBD level at second assessment

|  | Model 1 | Model 2 | Model 3 |
| --- | --- | --- | --- |
|  | β (95% CI) | β (95% CI) | β (95% CI) |
| Number of included participants | 1230 | 1230 | 1230 |
| Model constant | -33.4 (-38.0 – -28.8)** | -55.1 (-59.3 – -50.8)** | -54.3 (-59.7 – -48.9)** |
| lnBAU first sera assessment | -0.6 (-1.0 – -0.1)* | -1.1 (-1.5 – -0.8)** | -1.2 (-1.5 – -0.8)** |
| Days between first and second assessment |  | 0.4 (0.4 – 0.4)** | 0.48 (0.3 – 0.5)** |
| Long interval between assessments^#^ |  |  |  |
| >= 64 & <89 days |  |  | 2.7 (0.4 – 4.9)* |
| >= 90 days |  |  | 0.9 (-2.7 – 4.5) |
| Age in years | 0.1 (0.0 – 0.1)** | 0.0 (-0.0 – 0.1) | 0.0 (-0.0 – 0.1) |
| Female gender | 1.8 (0.5 – 3.2)* | 0.6 (-0.5 – 1.8) | 0.6 (-0.6 – 1.7) |
| R^2^ adjusted | 0.03 | 0.32 | 0.33 |
| AIC | 9535 | 9088 | 9077 |
| AIC: Akaike information criteria; lnBAU: logarithm of BAU value  Linear regression models with estimated percent change in anti-RBD during 30 days as outcome variable  ^#^Baseline comparator is <64 days between assessments  * p<0.05  ** p<0.001 | | | |

**Table S2** Median percentage change in anti-RBD level between first and second assessment across diagnoses

| **Diagnose** | **Percentage change** |
| --- | --- |
| RA % (IQR) | -80 (-91 **– -**62) * |
| PsA % (IQR) | -83 (-93 **–** -65) * |
| SpA % (IQR) | -86 (-94 **–** -75) * |
| UC % (IQR) | -84 (-97 **–** -62) * |
| CD % (IQR) | -86 (-96 **–** -70) * |
| *Percentage change in anti-RBD level compared between diagnoses and controls by Mann-Whitney U test, p<0.001  IQR=inter quartile range | |

**Table S3** Serological response at first and second assessment by medication group

|  | **First sera assessment** | **Second sera assessment** |
| --- | --- | --- |
| Controls (n=134)  anti-RBD <5, n (%)  anti-RBD 5**–**19, n (%)  anti-RBD 20**–**199, n (%)  anti-RBD 200**–**1999, n (%)  anti-RBD 2000**–**8999, n (%)  anti-RBD ≥ 9000, n (%) | 0  0  0  10 (7)  107 (80)  17 (13) | 0  0  6 (5)  70 (52)  58 (43)  0 |
| Methotrexate monotherapy (n=218)  anti-RBD <5, n (%)  anti-RBD 5**–**19, n (%)  anti-RBD 20**–**199, n (%)  anti-RBD 200**–**1999, n (%)  anti-RBD 2000**–**8999, n (%)  anti-RBD ≥ 9000, n (%) | 1 (0.5)  4 (2)  15 (7)  67 (31)  121 (55)  10 (4.5) | 6 (3)  12 (5)  45 (21)  113 (52)  42 (19)  0 |
| Interleukin inhibitors ^*^ (n=44)  anti-RBD <5, n (%)  anti-RBD 5**–**19, n (%)  anti-RBD 20**–**199, n (%)  anti-RBD 200**–**1999, n (%)  anti-RBD 2000**–**8999, n (%)  anti-RBD ≥ 9000, n (%) | 0  2 (5)  0 (0)  11 (25)  30 (68)  1 (2) | 0  3 (7)  5 (11)  27 (61)  9 (21)  0 |
| Vedolizumab (n=32)  anti-RBD <5, n (%)  anti-RBD 5**–**19, n (%)  anti-RBD 20**–**199, n (%)  anti-RBD 200**–**1999, n (%)  anti-RBD 2000**–**8999, n (%)  anti-RBD ≥ 9000, n (%) | 0  0  0  4 (13)  25 (78)  3 (9) | 0  0  2 (6)  16 (50)  13 (41)  1 (3) |
| Janus Kinase Inhibitors (n=22)  anti-RBD <5, n (%)  anti-RBD 5**–**19, n (%)  anti-RBD 20**–**199, n (%)  anti-RBD 200**–**1999, n (%)  anti-RBD 2000**–**8999, n (%)  anti-RBD ≥ 9000, n (%) | 0  0  0 (0)  9 (41)  11 (50)  2 (9) | 0  1 (4)  5 (23)  7 (32)  9 (41)  0 |
| TNFi monotherapy (n=464)  anti-RBD <5, n (%)  anti-RBD 5**–**19, n (%)  anti-RBD 20**–**199, n (%)  anti-RBD 200**–**1999, n (%)  anti-RBD 2000**–**8999, n (%)  anti-RBD ≥ 9000, n (%) | 0  0  17 (4)  156 (34)  273 (59)  18 (4) | 12 (2.5)  29 (6)  151 (32.5)  226 (49)  44 (9.5)  2 (0.5) |
| TNFi combination therapy^#^ (n=261)  anti-RBD <5, n (%)  anti-RBD 5**–**19, n (%)  anti-RBD 20**–**199, n (%)  anti-RBD 200**–**1999, n (%)  anti-RBD 2000**–**8999, n (%)  anti-RBD ≥ 9000, n (%) | 4 (1.5)  4 (1.5)  25 (9)  106 (41)  106 (41)  16 (6) | 20 (7.5)  26 (10)  101 (38.5)  95 (36.5)  19 (7.5)  0 |
| Rituximab (n=31)  anti-RBD <5, n (%)  anti-RBD 5**–**19, n (%)  anti-RBD 20**–**199, n (%)  anti-RBD 200**–**1999, n (%)  anti-RBD 2000**–**8999, n (%)  anti-RBD ≥ 9000, n (%) | 11 (35)  3 (10)  3 (10)  10 (32)  4 (13)  0 | 16 (52)  2 (6)  6 (19)  4 (13)  3 (10)  0 |
| Serological response is anti-RBD in BAU (binding antibody units/ml).  ^*^ tocilizumab, ustekinumab, risankizumab, secukinumab, iksekizumab  ^#^In combination with a metabolite inhibitor, prednisolone or vedolizumab  IQR=inter quartile range TNFi=tumor necrosis factor inhibitors JAKi= janus kinase inhibitors  Medication groups with less than 20 patients were not included in the table (n= 35 patients) | | |

| Variables | Model 1  β (95% CI) | Model 2  β (95% CI) | Model 3  β (95% CI) | Model 4  β (95% CI) |
| --- | --- | --- | --- | --- |
| **Demographics** | | | | |
| BMI kg/m^2^ | -0.0 (-0.1 – 0.1) |  |  |  |
| **Disease activity** | | | | |
| Patient global assessment |  | -0.0 (-0.1 – 0.0) |  |  |
| **Medication** | | | | |
| Methotrexate monotherapy |  |  | - | - |
| Interleukin inhibitors |  |  | -0.3 (-4.1 – 3.5) | -0.3 (-4.1 – 3.6) |
| TNF mono |  |  | -5.3 (-7.1 – -3.5) ** | -5.0 (-7.1 – -2.9) ** |
| TNF comb |  |  | -6.6 (-8.5 – -4.8) ** | -6.6 (-8.5 – -4.7) ** |
| Rituximab |  |  | 2.1 (-2.0 – 6.2) | 2.1 (-2.0 – -6.2) |
| **Diagnosis** | | | | |
| Rheumatoid arthritis |  |  |  | - |
| Psoriatic arthritis |  |  |  | 0.0 (-1.8 – 1.8) |
| Spondyloarthritis |  |  |  | -0.6 (-2.9 – 1.6) |
| ** p<0.001 *p<0.05  Linear regression models. Dependent variable of each model is estimated reduction in anti-RBD level in 30 days.  All models are adjusted for anti-RBD levels at first assessment and time between first and second sera assessment.  Please see table S3 for details.  Model 1 includes age, sex and body mass index  Model 2 includes age, sex, case vs. control and patient global assessment of disease activity  Model 3 includes age, sex, type immunosuppressive medication vs controls (Methotrexate monotherapy is comparator)  Model 4 includes age, sex, type immunosuppressive medication and diagnosis (Methotrexate monotherapy and rheumatoid arthritis are comparators) | | | | |

**Table S4** Linear regression models of estimated percent reduction in anti-RBD level in 30 days in patients with inflammatory joint diseases

**Table S5** Linear regression models of estimated percent reduction in anti-RBD level in 30 days in patients with inflammatory bowel disease

| Variables | Model 1  β (95% CI) | Model 2  β (95% CI) | Model 3  β (95% CI) | Model 4  β (95% CI) | Model 5  β (95% CI) |
| --- | --- | --- | --- | --- | --- |
| **Demographics** | | | | |  |
| BMI kg/m^2^ | -0.1 (-0.4 – 0.2) |  |  |  |  |
| **Disease activity** | | | | |  |
| Harvey Bradshaw index |  | -0.3 (-0.9 – 0.3) |  |  |  |
| Mayo score |  |  | 0.2 (-1.2 – 1.6) |  |  |
| **Medication** | | | | |  |
| Interleukin inhibitors |  |  |  | - | - |
| Vedolizumab |  |  |  | -0.3 (-7.5 – 6.9) | -0.2 (-7.5 – 7.2) |
| TNF mono |  |  |  | -13.0 (-19.2 – -6.9) ** | -13.0 (-19.2 – -6.8) ** |
| TNF comb |  |  |  | -9.8 (-16.5 – -3.0) ** | -9.7(-16.5 – -2.9) * |
| **Diagnosis** | | | | | |
| Ulcerative colitis |  |  |  |  | - |
| Crohn’s disease |  |  |  |  | 0.3 (-2.7 – 3.3) |
| ** p<0.001 *p<0.05  Linear regression models. Dependent variable of each model is estimated reduction in anti-RBD level in 30 days. All models are adjusted for anti-RBD levels at first assessment and time between first and second sera assessment. Please see table S3 for details.  Variables selected by forwards stepwise selection  Model 1 includes age, sex and body mass index  Model 2 includes age, sex, Harvey Bradshaw index in patients with ulcerative colitis  Model 3 includes age, sex, Mayo score in patients with Crohn’s disease  Model 4 includes age, sex, type immunosuppressive medication (Interleukin inhibitor is comparator)  Model 4 includes age, sex, type immunosuppressive medication and diagnosis (Interleukin inhibitor and rheumatoid arthritis are comparators) | | | | | |
